# Acute Intake of Fructose Increases Arterial Pressure in Humans: A Meta-Analysis and Systematic Review

**DOI:** 10.3390/nu16020219

**Published:** 2024-01-10

**Authors:** Sharif Hasan Siddiqui, Noreen F. Rossi

**Affiliations:** Department of Physiology, Wayne State University, 540 E. Canfield Ave. Scott 5473, Detroit, MI 48201, USA; hq4700@wayne.edu

**Keywords:** human, fructose, heart rate, blood pressure, glucose

## Abstract

Hypertension is a major cardiac risk factor. Higher blood pressures are becoming more prevalent due to changing dietary habits. Here, we evaluated the impact on blood pressure in human subjects after acutely ingesting fructose using meta-analysis. A total of 89 studies were collected from four different electronic databases from 1 January 2008 to 1 August 2023. Of these studies, 10 were selected that fulfilled all the criteria for this meta-analysis. Heart rate (HR), systolic blood pressure (SBP), diastolic blood pressure (DBP), mean arterial blood pressure (MAP), and blood glucose level were analyzed using the Cohen’s d analysis or standardized mean difference at a confidence interval (CI) of 95%. The SBP, DBP, and MAP showed medium effect size; HR and glucose level displayed small effect size. The standardized mean difference of normal diet groups and fructose diet groups showed a significant increase in SBP (*p* = 0.04, REM = 2.30), and DBP (*p* = 0.03, REM = 1.48) with heterogeneity of 57% and 62%, respectively. Acute fructose ingestion contributes to an increase in arterial pressure in humans. The different parameters of arterial pressure in humans correlated with each other. These findings support further rigorous investigation, retrospective of necessity, into the effect of chronic dietary of fructose in humans in order to better understand the impact on long term arterial pressure.

## 1. Introduction

Cardiovascular function plays a crucial role in healthy humans. Optimal cardiac function permits the body to meet the basic homeostatic demands including organ perfusion, oxygenation, and nutrition supply throughout the body as well as facilitates renal excretion of waste [1,2]. In recent years, people have become more concerned about modifiable factors that lead to cardiovascular diseases and increase cardiovascular mortality rates. There is an emerging body of evidence regarding the impact of fructose intake on cardiovascular function in preclinical and animal studies; however, there are few studies available in humans.

Cardiac function is associated with systemic parameters that can be easily measured clinically, including heart rate, blood pressure, and blood glucose level [3,4]. All these parameters should function well for proper cardiac function. Several factors contribute to the regulation of cardiac function such as the nervous system [5,6], hormones, environment [7] and food habits [8,9]. In this regard, food habits are among the modifiable factors. The intake of fructose has increased since 1970, with the addition of high-fructose corn syrup to several foods, food additives, and beverages [6]. High-fructose diets increase blood pressure by enhancing renal sodium and chloride transport as well as the excitation of the sympathetic nervous system and dysregulation of vasoconstrictors and vasodilators [10,11,12,13]. Moreover, high fructose intake increases oxidative stress and activates the renin –angiotensin–aldosterone system, which contributes to high blood pressure [10,14,15,16,17,18]. A previous study demonstrated that adolescents are more sensitive to fructose [19]. Moreover, it is alarming that fructose consumption is increasing on account of increasing trends in consumption of processed food and sugared beverages that contain high fructose [20]. According to the United States Department of Agriculture (USDA), fructose intake has continuous increased with an average fructose intake of 37 g/day in 1977 but 49 g/day in 2004 [21]. The fructose contents of foods and condiments promotes obesity and has an independent effect on hypertension, glucose intolerance and fatty liver diseases [22].

However, the impact of fructose on cardiac function is still unclear because only limited research has been conducted on fructose and cardiovascular function in humans. Therefore, in this study, we discuss the relationship between a single calibrated dose of fructose intake and blood pressure and blood glucose levels in humans. Most existing studies examining the role of fructose on blood pressure in humans have used dietary recall, food frequency questionnaires, and short-term diet records, which are subject to variability or bias [23,24]. Studies of consumption of common sugar-sweetened beverages contain varying amounts of fructose and often studies report only the number of such beverages and not the volume of fluid ingested [25,26]. Appraisal of existing studies about the relationship between cardiovascular function and fructose may provide insights and point to areas fruitful for further investigation. Thus, we embarked on a meta-analysis about blood pressure and fructose intake in human subjects.

In this study, we performed a meta-analysis to evaluate the impact on blood pressure and blood glucose levels in humans after receiving a fructose load during different treatment periods. We list the statistical analyses conducted to determine the relationship between different parameters of arterial pressure and fructose after fructose intake. To the best of our knowledge, this is the first meta-analysis that addresses the comparative arterial pressure of humans after fructose ingestion.

## 2. Materials and Methods

This systematic review and meta-analysis were conducted following the Preferred Reporting Items for Systematic Reviews and Meta-Analyses (PRISMA) criteria (Table S1).

### 2.1. Search Strategy

We searched previously published articles using different electronic databases. The clinical studies were collected from PubMed, Web of Science, Cochrane Library, and Em-base from 1 January 2008 to 1 August 2023. The selected articles must have been published as research articles. All the studies must have evaluated humans. The literature search keywords were human, heart rate (HR), systolic blood pressure (SBP), diastolic blood pressure (DBP), mean arterial pressure (MAP), glucose, and fructose. The titles and abstracts of the enrolled articles were obtained by searching the selected keywords following the selection conditions.

### 2.2. Selection Criteria

**Figure 1:**
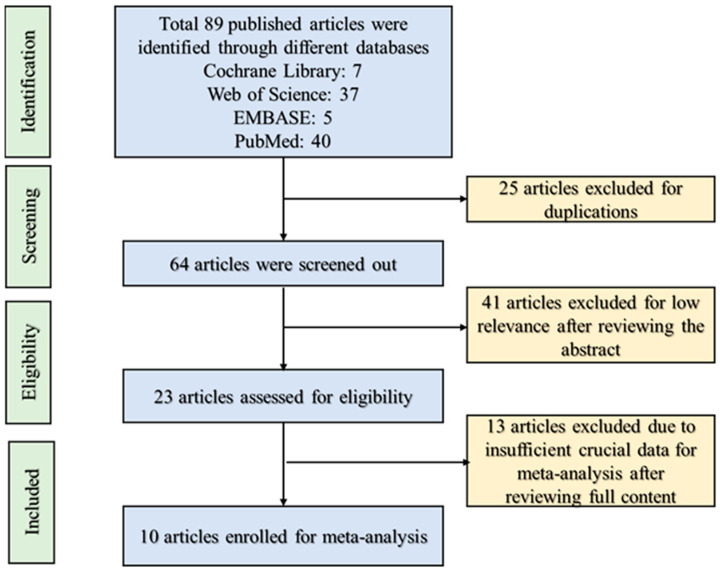
The study selection flowchart. Randomized controlled trials published before September 2023 in the PubMed, Web of Science, EMBASE, and Cochrane library databases were searched succeeding the Preferred Reporting Items for Systematic Re-view and Meta-Analysis (PRISMA) guidelines.

The selected articles were assessed on the basis of their relevance and consequences. The selection method for published articles is demonstrated in a flowchart (Figure 1). The articles were selected for this meta-analysis when they fulfilled the following conditions:

1. Studies should include human subjects.
2. Only studies evaluating acute fructose loads were included.
3. The selected articles must have a normal diet group for comparison with the fructose diet group.
4. The articles should have two parts: the starting and ending points of fructose in-take humans.
5. The data addresses regulation of prediabetic and diabetic conditions as well as hypertension.
6. All the selected data should be presented in either tabulated form or figure with the standard deviation (SD) or standard error (SE).
7. The data of the selected articles must be well organized and described. Humans must be free from pre-existing metabolic and cardiovascular diseases.
8. Subjects should be nonalcoholic.
9. The selected articles must be published in an international peer-reviewed journal within the selected study period. This indicates that preprint or unpublished articles were not considered for this study. Moreover, all selected articles must be written in English.

Studies with acute raw fruit intake were not included since they contain other ingredients, such as flavonoids, carotenoids, epicatechin, lycopene, ascorbic acid and others [27], which may lower blood pressure. In addition, investigations evaluating sucrose ingestion were excluded since sucrose is a dimer of glucose and fructose that may have dual or opposing effects on metabolism and other pathways.

### 2.3. Study Classification

A total of 10 appropriate studies were selected from 89 primary peer-reviewed articles because they fulfilled all the conditions. Each article was evaluated in terms of fructose intake during the experiment. We considered two different groups: (i) the normal food habit group and (ii) the fructose intake group. In addition, duplicate studies identified by the databases were recognized, and we selected the most up-to-date article that more comprehensively addressed the study criteria listed above.

### 2.4. Data Extraction

We extracted the data from the enrolled articles for this study individually using a pre-designed related questionnaire that considered different research areas. We did not extract the data from the figure directly but used the Plot Digitizer v.3 software (http://plotdigitizer.sourceforge.net/ (accessed on 14 September 2023)). To provide a rigorous meta-analysis, we considered the following information for this study: data source (authors and publication year), history of pre-existing conditions (disease condition, food habit, alcoholic, smoking consent for study), study design, sample size, data extraction (HR, SBP, DBP, MAP, and glucose), and clear description of the data. We considered clinical (human) data independently for this study. We measured variable accuracy by assessing risk. Nevertheless, we recognized that different food habits of humans prior to the study may exert effects on body condition and function. For this study, we used Kappa coefficient analysis to assess the level of agreement between the control and fructose diet in humans as well as to understand the correlation coefficient [28]. For example, the cast-off Kappa coefficient was used to analyze data consistency between different groups. This analysis assists in measuring the same conditions between the normal diet and fructose diet groups. Furthermore, this analysis showed a correlation between the normal diet and fructose diet groups (correlation coefficient =  0.99; 95% confidence interval [CI]: 0.99 and 0.99). Thus, we are confident that our selected articles have low bias for data extraction for this meta-analysis [29].

### 2.5. Study Quality Assessment

The selected articles were evaluated according to a previous study [30] using the Physiotherapy Evidence Database (PEDro) scale; however, the article selection criteria of this study are not related to that scale. Nonetheless, this scale helps select effective articles for the meta-analysis and helps perform statistical analysis of the data from the selected articles [31]. This scale evaluates the quality of the selected articles using 11 criteria that are aligned with the experimental design. The PEDro scale ranges from 0 to 10. High-quality articles obtain a score ≥ 7, medium quality is scored 5–6 and a ≤4 score represents poor-quality articles [32].

### 2.6. Statistical Analysis

Statistical analysis was performed using R version 4.3.1 (Vienna, Austria: the R Foundation for Statistical Computing). The aim of this study was to compare the different parameters of blood pressure before and after fructose consumption. The random effects model (REM) with a 95% CI and odds ratio was used to evaluate the effect size of persistence and dichotomous results, respectively. Heterogeneity was calculated for the selected articles using Cochran’s Q statistic, and the I2 test and the bias of the selected articles were analyzed using a funnel plot. We considered heterogeneity in this study when *p* = 0.05 and I2 was 50. Begg’s test was used to analyze bias in the publication of the selected articles [33]. This meta-analysis detected low-to-moderate heterogeneity response elements, so a random effects model was selected for the article selection procedure and the detection of variation in these studies [34]. The standardized mean difference between normal and fructose diet was analyzed using the meta package (https://cran.r-project.org/web/packages/meta/index.html (accessed on 14 September 2023)), and Cohen’s d analysis was conducted using effsize (https://cran.r-project.org/web/packages/effsize/index.html (accessed on 14 September 2023)). We conducted correlation co-efficient and PCA analysis using the ggplot2 package (https://cran.r-project.org/web/packages/ggplot2/index.html (accessed on 14 September 2023)).

## 3. Results

### 3.1. Study Retrieval and Selection

**Figure 2:**
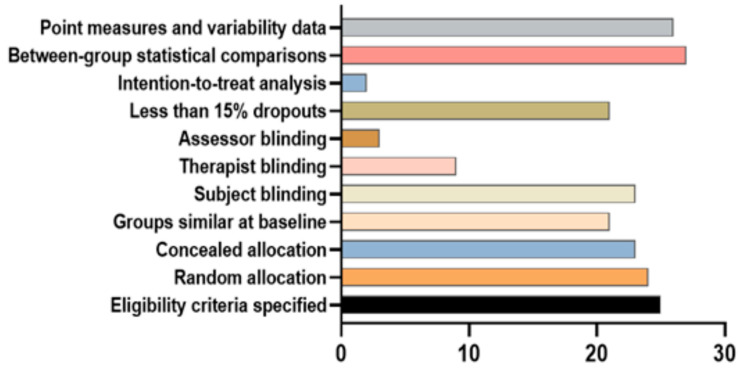
Number of studies undergoing individual PEDro [Physiotherapy Evidence Database] conditions.

The article search and selection procedure are shown in Figure 1. A total of 89 articles were extracted from four different electronic databases. We excluded 25 articles from the primary list for duplication among the databases. We excluded 41 articles due to a lack of relevancy and insufficient crucial data for this meta-analysis. Assessment of the quality of the selected articles was conducted using the PEDro scale (Figure 2). The selected articles of this meta-analysis are of moderate quality. Ultimately, 10 articles were selected because of their suitable data for the hypothesis of this study. The characteristics of the selected articles are presented in Table 1. The PEDro scale criteria fulfilled by each selected article are provided in Table S2.

### 3.2. The Effect Size of Fructose on Blood Pressure, Heart Rate and Blood Glucose Level

The effect size of fructose was analyzed via Cohen’s d analysis with a 95% confidence interval (CI). After the fructose diet, the effect size of SBP, DBP, and MAP was medium. The effect size of HR and blood glucose levels is small. The value of Cohen’s d of SBP, DBP, MAP, HR, and blood glucose levels was 0.587, 0.642, 0.7063, 0.473, and 0.216, respectively (Table 2). These results reveal that the fructose diet does impact blood pressure, heart rate, and blood glucose levels.

### 3.3. Analysis of Study Standariezed Mean Difference and Bias Levels

**Figure 3:**
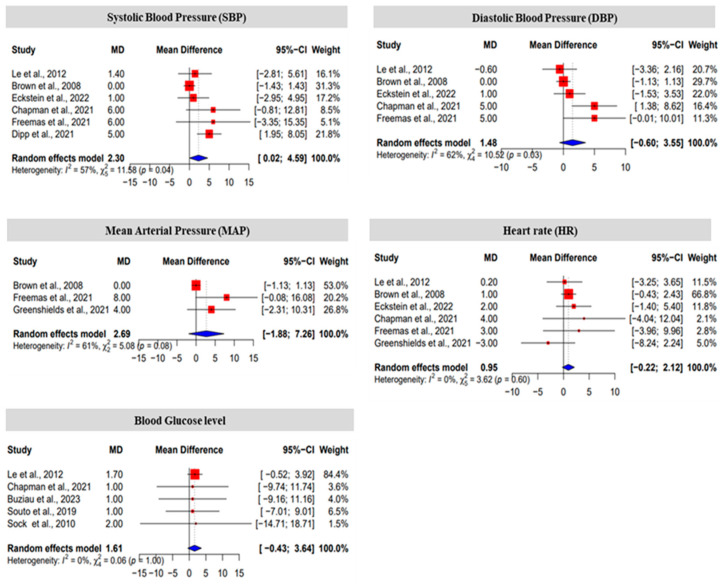
Forest plots represent the effect of fructose on systolic blood pressure (SBP), diastolic blood pressure (DBP), mean arterial blood pressure (MAP), heart rate (HR), and blood glucose level of humans using standardized mean difference. Red, mean difference of individual study; blue, mean difference for the random effects model [20,32,35,36,37,38,39,40,41,42,43].

**Figure 4:**
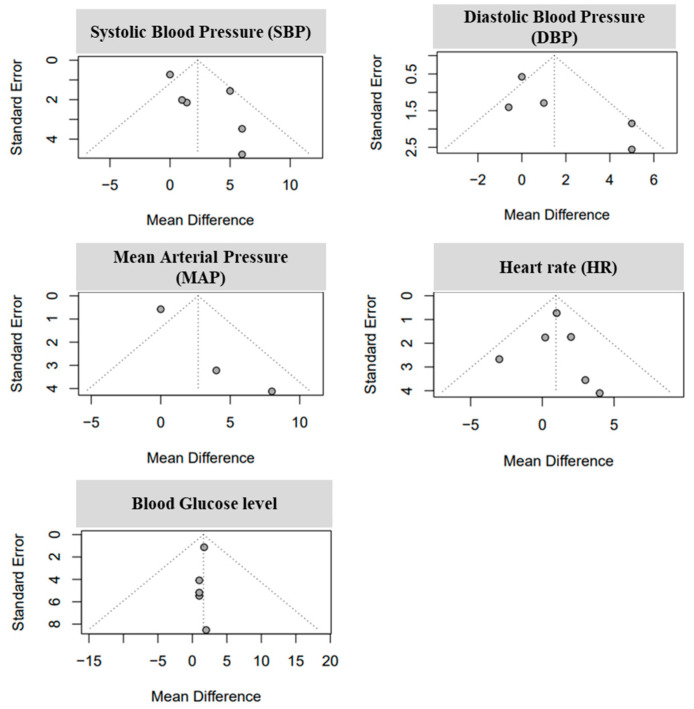
Funnel plots representing the effect of fructose on hypertension of human-related published journal bias selected for meeting criteria for this study. The points indicate individual studies within the funnel plots.

Among the 10 articles analyzed, heterogeneity was not found in HR or glucose levels. However, the heterogeneity of SBP, DBP and MAP was significant in the normal and fructose diets, displaying a heterogeneity of 57%, a random effect model (REM) = 2.3, and *p* = 0.04 for SBP; a heterogeneity of 62%, a REM = 1.48, and *p* = 0.03 was found for DBP; and MAP showed a 61% heterogeneity, REM = 2.69, and *p* = 0.08 (Figure 3). We analyzed the publication bias of the selected articles using funnel plots. The funnel plots show that all the selected articles were asymmetrical, which indicates that there was a publication bias, except for HR and blood glucose in the clinical trials (Figure 4).

## 4. Discussion

Dietary recall surveys or correlative data from general trends in dietary intake and pathophysiologic findings in populations provide intriguing information but are not sufficient for a better understanding of the direct effect of fructose itself on blood pressure in humans. Unfortunately, our search for studies in human subjects was only able to identify controlled studies reporting the acute effects of acute fructose ingestion (several hours). In contrast, animal studies showing deleterious effects on hemodynamics have largely assessed the long-term effects (weeks to months) of fructose on blood pressure [44,45,46,47], with some animal studies using dietary amounts exceeding what is typically ingested by people [36,37,38,39]. We identified only a limited number of publications reporting the direct effects of fructose on blood pressure in humans. Furthermore, the existing studies have very small sample sizes, limiting the power of individual analyses. Consequently, we evaluated the effect of acute fructose ingestion on blood pressure in human subjects using meta-analysis. Our meta-analysis clearly demonstrates that within 1–6 h of a fructose load, levels are consistent with amounts comparable to the intake of a single serving of commercially available fructose-containing beverages, significantly increases systolic, diastolic, and mean blood pressures.

Since hypertension has a significant impact on cardiovascular health and poses the single greatest risk for deleterious outcomes, fructose-rich food consumption’s propensity to increase blood pressure raises the risk of major adverse cardiovascular events and mortality [48]. Moreover, blood pressure parameters directly impact cardiovascular function, such that elevations of blood pressure, even within the normal range, may contribute to an increased risk of cardiovascular disease [49,50,51,52,53,54,55,56]. Despite its low glycemic index, fructose intake has been associated with lipogenesis, insulin resistance, and type 2 diabetes mellitus. In the long term, increases in lipid deposition in the blood vessels may result in chronic elevations in blood pressure and the risk of heart disease [57]. It has been reported that therapeutic approaches to type 2 diabetes that relieve oxidative stress and modulate lipid metabolism decrease the risk of cardiovascular disease [58,59]. Oxidative stress also contributes to insulin resistance and vascular dysfunction with fructose intake [60]. Interestingly, sucrose exerts minimal influence on oxidative stress despite the fact that insulin sensitivity is reduced in rats [61]. 

In exercising humans, when fructose is consumed with glucose either as individual monosaccharides or as disaccharide sucrose in beverages or foods, carbohydrate oxidation rates increase [62]. In preclinical studies, interventions to limit oxidative stress ameliorate fructose-induced elevations in blood pressure as well as indices of insulin resistance [17]. This highlights a key difference in the consumption of fructose in raw fruit rich in antioxidants versus beverages, condiments, and other foods supplemented with high-fructose corn syrup [27].

The current meta-analysis attempts to identify studies that isolated the effect of fructose from food items that could mitigate or exacerbate the impact of a fructose load. For example, some vitamins and minerals independently lower blood pressure. These studies have been evaluated in a separate meta-analysis by Behers et al. [63]. In real life, the influence of concurrent intakes of various nutrients is complex and may account for some of the disparate results seen in chronic studies.

There are several mechanisms whereby fructose intake can adversely affect cardiovascular function. The consumption of fructose alters lipoprotein distribution [48], which, in turn, enhances the deposition of small and dense LDL particles [64]. This particle deposition increases the risk of cardiovascular disease [65] due to the formation of atherosclerotic plaque [66]. Fructose intake does not suppress satiety as effectively as glucose since fructose intake results in lower levels of glucose, insulin, leptin and ghrelin compared with a similar caloric intake of glucose [67]. Moreover, long-term fructose consumption induces insulin resistance, hypertriglyceridemia, and lipid accumulation in the liver, leading to nonalcoholic fatty liver disease and the development of type 2 diabetes [68]. Notably, fatty acid production is augmented by fructose but not glucose or sucrose [69]. In so doing, high-fructose consumption promotes lipogenesis and triglyceride (TG) accumulation in the liver, contributing to insulin resistance [70] and type 2 diabetes mellitus [71]. There are several pathways responsible for hepatic insulin resistance, such as endoplasmic reticulum (ER) stress, hepatic lipogenesis, mitochondrial dysfunction, and liver inflammation [72]. In addition, the long-term consumption of fructose activates the JNK pathway or unusual lipid metabolism, which induces hepatitis, thus stimulating inflammatory cells that produce proinflammatory cytokines that augment insulin resistance [72]. Fructose intake promotes hepatic VLDL-TG secretion, which fosters hypertriglyceridemia [73]. Thus, fructose further increases lipid accumulation by altering lipid and carbohydrate metabolism [74] and lipid accumulation, eventually resulting in nonalcoholic fatty liver disease [75]. Moreover, the intake of fructose-sweetened beverages increases visceral adiposity, a known risk factor for cardiovascular disease. In contrast, despite comparable caloric intake and weight gain, the consumption of glucose-sweetened beverages over a 10-week period does not increase visceral adiposity [76]. In addition, fructose promotes inflammation and lipid accumulation in cardiac myocytes and enhances myocardial and vascular superoxide generation [77,78]. Together, these mechanisms conspire to promote hypertension and adverse cardiovascular effects.

Whether insulin resistance precedes or results from vascular dysfunction has been debated [79]. Notably, the subjects included in this meta-analysis were, by design, not diabetic or hypertensive. We can speculate that the blood pressure effects may be accentuated in individuals ingesting repeated fructose loads over time or with these pre-existing conditions [80] or who already have activated inflammatory pathways, as evidenced by the hypotensive effect of anti-inflammatory medications [81]. In addition, studies in rats show that a diet containing 20% weight/volume fructose attenuates aortic relaxation via nitric oxide, whereas a similar glucose-rich diet augmented vasodilation [82]. Similar findings have been observed in resistance vessels [83] but diminished vasodilatory capacity could also contribute to fructose-induced elevations in blood pressure. Hypertension may be further exacerbated since fructose appears to impart salt sensitivity, meaning that urinary sodium excretion is impaired with fructose intake of roughly 20% of total calories [12,84]. High salt intake increases endogenous hepatic fructose generation [85]. Since, in real life, high fructose beverages are often ingested together with food items high in sodium content, the impact of fructose consumption on a chronic day-to-day basis may be further amplified in the propensity to develop hypertension.

In the modern world, people are becoming more dependent on eating canned or processed food. These foods provide high energy as they contain a large amount of fructose, particularly in the form of high-fructose corn syrup. Previous studies reported that a 60 g fructose drink acutely increases heart rate as well as blood pressure [36] by suppressing β-adrenergic activity [86]. Our meta-analysis is consistent with these findings in that fructose increases heart rate as well as systolic, diastolic, and mean blood pressure. In addition, our results are consistent with studies that revealed that chronic fructose consumption increases systolic and diastolic blood pressure [19,22]. Preclinical studies strongly suggest that the effect of fructose on cardiac function may be further influenced by the dose and duration of fructose intake. For instance, a high dose of fructose (60% of caloric intake daily) consumption for 20–28 days remarkably increases mean arterial pressure in dogs [87]. Moreover, consumption of a 60% fructose diet in rodents decreases left ventricular ejection fraction [88]. Rodents fed 20% fructose as their daily caloric intake, more consistent with the fructose intake in the upper quintile of human subjects, did not display left ventricular diastolic dysfunction, but systolic left ventricular function remained unaffected [89,90]. Notwithstanding, these changes occur despite normal blood glucose levels in the fructose-fed rats and do not elevate blood glucose levels [12,91]; however, insulin resistance was evident despite euglycemia [11,18,90]. Furthermore, a fructose-rich diet is associated with increased aortic stiffness in both rats [47,90] and humans [92]. Aortic stiffness is now recognized as a significant risk factor for cardiovascular disease and mortality [93,94]. Importantly, a recent meta-analysis of drinks sweetened with non-nutritive, FDA-approved additives, such as aspartame, stevia, sucralose, acesulfame potassium, monk fruit extract or advantame, show that such beverages do not provoke metabolic and endocrine effects that are seen with fructose [95]. Existing data strongly support urging individuals to substitute water or such non-nutritive sweetened beverages as alternatives to drinks supplemented with high-fructose corn syrup and other fructose-containing additives.

### Limitations

Thus, our meta-analysis of the impact of acute human fructose ingestion on hemodynamic parameters is consistent with earlier cross-sectional studies [96,97]. Nonetheless, one limitation is that the number of selected articles meeting the stringent criteria was low; thus, stratification by race, age, and sex was not possible. In addition, fructose exposure was after only one treatment and of short duration (hours). Unfortunately, chronic controlled trials are neither feasible nor would they be ethical given the current understanding of the impact of fructose on health. Nonetheless, more rigorously designed studies, such as interrogations of meticulously recorded food diaries to evaluate the impact of chronic high fructose intake or repeated acute intake of fructose foods on blood pressure and cardiovascular parameters in humans, are warranted.

## 5. Conclusions

Fructose affects blood pressure in humans by regulating different factors of the cardiovascular system. The present meta-analysis focused on the effect of acute fructose consumption on blood pressure. Consequently, further rigorous chronic studies of humans, likely utilizing detailed database data with verified dietary intakes, are needed to better understand the impact of fructose on hemodynamics and cardiovascular function in humans. It is nonetheless prudent to consider efforts to limit dietary fructose intake.

## Figures and Tables

**Table 1:** Characteristics of selected publications.

SI	Authors [Ref]	Sample Size (*n)*	Experiment Duration	Treatment (Fructose)	Experimental Condition
1	Le et al., 2012 [35]	40	6 h	24 oz of cold, carbonated soft drinks sweetened with high fructose	- Alcohol consumption was prohibited 3 h prior to the experiment - Prior to study, minimum 8 h fasting - Exercise was prohibited - Not given additional calories during the experiment
2	Brown et al., 2008 [36]	15	2 h	60 g fructose (water)	- Alcohol and caffeine consumption was prohibited 24 h prior to the experiment - Prior to study, minimum fasting - Empty their bladder immediately prior to the experiments
3	Eckstein et al., 2022 [37]	15	2 h	50% fructose in drinking water	- The participants were overnight fasted - The participants were free from abnormal blood pressure and metabolic diseases - Alcohol, caffeine, and soda consumption was prohibited 24 h prior to the experiment - Exercise was prohibited during the fasting period
4	Chapman et al., 2021 [38]	12	2 h	Soft drinks contain 50% fructose	- The participants were overnight fasted - Alcohol and caffeine consumption was prohibited 24 h prior to the experiments - Exercise was prohibited during the fasting period
5	Freemas et al., 2021 [39]	13	2 h	500 mL Mountain Dew which contains 59.5% fructose	- Participants were instructed to avoid caffeine, energy drinks and alcohol 12 h prior to the experiments - Exercise was prohibited 12 h prior to the experiments - Food was restricted for 2 h prior to the experiments
6	Greenshields et al., 2021 [32,40]	14	2 h	500 mL Coca Cola	- The participants were physically healthy - The participants were nonsmokers and nonalcoholic - The participants were free from cardiovascular, neurological, and metabolic diseases
7	Dipp et al., 2021 [41]	14	1 h	Drink contains 100 g fructose, 30 mL lemon juice in 300 mL water	- The participants were physically active and diseases free - The participants were nonsmokers and nonalcoholic
8	Buziau et al., 2023 [42]	13	2 h	82.5 g dextrose monohydrate and 15 g fructose dissolved 250 mL water	- The participants should sleep well - Prior to study, minimum overnight fasting
9	Souto et al., 2019 [43]	7	3 h	75 g fructose dissolved with 200 mL water	- The volunteer should be free from diseases - Alcohol and caffeine consumption was prohibited 24 h prior to the experiment
10	Ngo Sock et al., 2010 [20]	11	1 h	3.5 g fructose per kg of meal	- The volunteers were physically active - The volunteers were nonsmokers - The volunteers were 10 h fasted prior to the experiments

**Table 2:** Table of Cohen’s d effect sizes and corresponding 95% confidence intervals (CI).

Parameter	95% Confidence Interval (CI)		Cohen’s d	Effect Size *
	**Lower**	**Upper**		
Systolic Blood pressure (SBP)	−0.726	1.901	0.587	medium
Diastolic Blood Pressure (DBP)	−0.853	2.137	0.642	medium
Mean Arterial Pressure (MAP)	−2.284	3.697	0.706	medium
Heart Rate (HR)	−0.614	1.56	0.473	small
Blood Glucose Level	−1.518	1.951	0.216	small

* Cohen’s d effect size weighted by degrees of freedom.

## Data Availability

All study data are provided in the manuscript and Supplementary Materials. The data are from already existing data from published articles available in the public domain. No new data were created. Methodology used for the systematic analysis is provided in the manuscript. Methods and data are available upon request from the corresponding author.

## Supplementary Materials

The following supporting information can be downloaded at: https://www.mdpi.com/article/10.3390/nu16020219/s1, Table S1: PRISMA 2020 Checklist; Table S2: The score of PEDro scale of each selected article. Ref. [98] is cited in the supplementary materials.
